# Supplementary material for: Risk factors associated with nosocomial infections among end stage renal disease patients undergoing hemodialysis: A systematic review
Source: PLoS One. 2020 Jun 22;15(6):e0234376. doi: 10.1371/journal.pone.0234376 (PMC7307739; doi:10.1371/journal.pone.0234376)
Supplement: Attachment — Submitted filename: Response to reviewers.docx [file pone.0234376.s002.docx]

Aries Systems Corporation


Coronavirus (COVID-19) Update from Aries Systems

# Aries Systems Corporation

- Request a Demo
- Contact Us

Toggle navigation

- Solutions
  - Solutions
  - Editorial Manager
  - LiXuid Manuscript
  - ProduXion Manager
  - Task Manager
  - Commerce Manager
  - Standards
- Journals List
- Support & Resources
  - Client Services
  - Resources
  - User Community
- Videos
- Views & Press
  - News & Opinion
  - Press Releases
  - Newsletter Archive
- Events
- About
  - About Aries
  - Partners
  - Testimonials
  - Privacy Policy
  - Careers

- Home
- Solutions
  - Solutions
  - Editorial Manager
  - LiXuid Manuscript
  - ProduXion Manager
  - Task Manager
  - Commerce Manager
  - Standards
  - User Community
- Journals List
- Support & Resources
  - Client Services
  - Resources
  - Videos
  - User Community
- Videos
- Views & Press
  - News & Opinion
  - Press Releases
  - Newsletter Archive
- Events
- About
  - About Aries
  - Partners
  - Testimonials
  - Privacy Policy
  - Careers
- Request a Demo
- Contact Us
- Careers

# Publish Faster. Publish Smarter.

## Workflow infrastructure solutions for scholarly publishers

Manuscript Submission   
& Peer Review

Comprehensive XML   
Workflow Solution

Manuscript Production   
& Task Tracking

View All Solutions

## Videos

Browse hundreds of videos showcasing Editorial Manager and ProduXion Manager system features and functionality

Watch Videos

## Journals List

Looking for a specific journal using Editorial Manager? Search our Journals List to locate a publication’s EM/PM portal

See Journals List

## Testimonials

Don’t take our word for it. See what current users are saying about their experience with Editorial Manager and ProduXion Manager

Read Reviews

## FAQs

Have a question? Find answers to our most common queries about access and support, GDPR, adopting a new system and more!

View FAQs

## You’re in Good Company

## Recent News & Press

## Meet the Aries Team: Brenda Dean, Software Engineer 4

## Aries Systems Announces Partnership with AAMC for Streamlined Disclosure Workflow Solution for Publishers

## Free Webinar – Streamlining OA Workflows: Use Matadata to Manage APCs with Copyright Clearance Center

## Aries Systems and The Charlesworth Group Partner to Enable Access to Editorial Manager^®^ through the Chinese Social Media App WeChat

View All Press Releases
View All News & Opinion


## Looking for More Information?

We’re happy to answer questions and provide free demonstrations.

Request a Demo

- Privacy Policy
- Japanese Version

- Aries Systems Corporation
- 50 High Street, Suite 21
- North Andover, MA, 01845

© 2020 Aries Systems Corporation. Editorial Manager, LiXuid Manuscript, ProduXion Manager, and Commerce Manager are trademarks and the property of Aries Systems Corporation. All Rights Reserved. Subject to change without notice from or liability to Aries Systems Corporation.
